# The Ancient Drug Salicylate Indirectly Targets Fructose‐1,6‐Bisphosphatase to Suppress Liver Glucose Production in Diet‐Induced Obese Mice

**DOI:** 10.1111/apha.70058

**Published:** 2025-05-22

**Authors:** Raid B. Nisr, Abdelmadjid Atrih, Erika J. Gutierrez Lara, Douglas Lamont, Katarzyna M. Luda, Rory J. McCrimmon, Kei Sakamoto, Graham Rena, Alison D. McNeilly

**Affiliations:** ^1^ Division of Diabetes, Endocrinology and Reproductive Biology, School of Medicine University of Dundee Dundee UK; ^2^ Centre for Advanced Scientific Technologies, School of Life Sciences University of Dundee Dundee UK; ^3^ Novo Nordisk Foundation Center for Basic Metabolic Research University of Copenhagen Copenhagen Denmark

**Keywords:** fructose‐1,6‐bisphosphatase, glucose, liver, obesity, salicylate

## Abstract

**Aims:**

The benefit of salicylate in the treatment of diabetes has been recognized for over a century; however, challenging side effects have prevented widespread use. A better understanding of the relevant enzyme targets mediating its anti‐hyperglycaemic effect may lead to the development of novel therapies for diabetes. Here, we investigated the contribution of 5′‐adenosine monophosphate (AMP)‐dependent inhibition of fructose‐1,6‐bisphosphatase 1 (FBP1) to the anti‐hyperglycaemic action of salicylate.

**Methods:**

We studied AMP‐insensitive FBP1 G27P knockin (KI) mice through a variety of cellular approaches, including proteomics, Seahorse metabolic analysis, glucose production, and other assays, in addition to a detailed assessment of metabolic responses in vivo.

**Results:**

Compared with wild‐type littermates, AMP‐insensitive FBP1 KI mice were resistant to the effects of the drug on body weight, glucose tolerance, pyruvate disposal, liver lipid content and hepatic glucose production. Compared with wild‐type, KI hepatocytes exhibited baseline differences in glycolytic, TCA cycle and fatty acid oxidation enzyme levels, potentially linking gluconeogenic dysregulation and its reversal to non‐carbohydrate fuel management.

**Conclusion:**

Collectively, our data highlight a novel mechanism of action for the effects of salicylate on glycaemia and weight gain, which depends on AMP‐mediated allosteric inhibition of FBP1.

## 1 Introduction

Empirical therapeutic use of natural salicylate‐containing plants has been ongoing since at least the early days of civilization in most parts of the world [1], and possibly encompassing near hominin relatives [2]. Attempts to exploit pure salicylate for use in diabetes were first reported around 150 years ago [3, 4]; however, it has never achieved clinical use owing to challenging side effects, including tinnitus and gastric bleeding [5]. Exploitation of the antidiabetic effect of salicylate with other chemical structures has not been possible either because of continuing uncertainty about the relevant protein target(s). Mice are a model of obesity‐induced type 2 diabetes amenable to genetic modification [6]. Although knockouts of inflammatory genes mimic antihyperglycaemic effects of salicylate [7, 8], there has been no genetic targeting of an inflammatory pathway that ablates the effect of salicylate on gluco‐regulation, and we have found that inhibitors of inflammatory signaling do not affect hepatic glucose production [9]. Mutation of the salicylate binding site on AMPK [10] does not ablate the antihyperglycaemic effect of the drug either [11]. More recent studies have re‐evaluated the importance of mitochondrial respiratory inhibition, which was one of the earliest models proposed to explain the anti‐hyperglycaemic effect of the drug [9, 11].

Recent work in mice has identified AMP‐dependent regulation of fructose‐1,6‐bisphosphatase 1 (FBP1) as a critical indirect anti‐hyperglycaemic target of the most widely used diabetes drug metformin [12]. Metformin and salicylate are both inhibitors of mitochondrial ATP production [9, 11, 13], and they both target hepatocyte glucose production [9, 14]. These common features prompted us in the current study to investigate, through cellular and in vivo measurements, the hypothesis that salicylate acts on FBP1 to mitigate dysglycaemia in the context of a high fat diet (HFD).

## 2 Materials and Methods

### 2.1 Chemicals and Reagents

Dulbecco's Modified Eagle's Medium (10569010), HBSS (15266355), collagenase (17104019), Earle's Balanced Salt Solution (EBSS) (11540616), MEM Amino Acids 50X (11130051), glutamine (25030024), sodium pyruvate (11360070), Pierce 16% Formaldehyde (10751395) came from Fisher. Metformin (317240), oligomycin (O48765), FCCP (carbonyl cyanide p‐trifluoromethoxy phenylhydrazone) (C2920), rotenone (45656), antimycin‐A (A8674), BSA (bovine serum albumin) (A8412), triiodothyronine (Sigma T2877), dexamethasone (265005), lactic acid (L1750), glucagon (G2044), DMEM glucose, glutamine and phenol red free (D5030), salicylic acid (S3007), collagen from bovine skin (C2124) came from Merck. Insulin Actrapid was from Novo Nordisk (041–7642).

### 2.2 Cell Culture and Immunoblotting

Primary hepatocytes were extracted from wild‐type (WT) and FBP1 knockin (KI) mouse livers by collagenase digestion as described previously [15]. Male and female mice were used for all in vitro studies. Briefly, pelleted hepatocytes were resuspended in DMEM supplemented with 100 μg/mL penicillin, 100 μg/mL streptomycin, 0.1% (v/v) BSA, 10% FBS, 10 nM insulin, 200 nM triiodothyronine (Sigma T2877), and 500 nM dexamethasone (Merck 265 005). Cell viability and number were measured using a hemocytometer with cells stained with 0.04% trypan blue stain. Cell viability of greater than 90% was required for experimental use. After isolation, cell culture plates were coated with gelatin (0.2%) for 20 min at 37°C. Cultures were maintained at 37°C and 5% CO_2_ for 4 h, and media were replaced with DMEM 0.5% FBS and used the following day. Hepatocytes were washed twice in warm PBS before treatment with salicylate or metformin. When studying mTOR activity, primary hepatocytes were serum and amino acid starved in EBSS medium for 2 h and then refed with MEM amino acids for 1 h with and without salicylate doses of 1, 2, 5, and 10 mM.

For immunoblotting, cells were lysed by scraping into ice‐cold buffer A (50 mM Tris acetate pH 7.5, 1% (w/v) Triton X‐100, 1 mM EDTA, 1 mM EGTA, 0.27 M sucrose, 50 mM NaF, 1 mM sodium orthovanadate, 10 mM β‐glycerophosphate, 5 mM sodium pyrophosphate, 1 mM benzamidine, 0.2 mM phenylmethylsulfonyl fluoride (PMSF), and 0.1% (v/v) β‐mercaptoethanol). Protein concentration was measured with Bradford reagent and BCA. The lysates were subjected to SDS‐PAGE in 12% pre‐cast gels and then transferred to PVDF membranes pre‐soaked in absolute methanol. Membranes were then probed for specific primary antibodies. Antibodies supplied by Cell Signaling Technology were (used 1:1000) pAMPKα‐Thr172 (2535S), AMPKα (2532S), pACC‐Ser79 (3661S), and ACC (3676S), p‐p70S6K Thr389 (9234S), p70S6K (2708S), p‐S6 Ser240/244 (2215S), and S6 (2317S). Following primary antibody incubation, detection was performed using secondary mouse (#7076S) or rabbit (#7074S) horseradish peroxidase (HRP) conjugated antibodies from Cell Signaling Technology and visualized using enhanced chemiluminescence (Pierce‐Perbio Biotech, Tettenhall, UK) on Kodak X‐OMAT film (Eastman‐Kodak, Rochester, UK). The immunoreactive protein bands were quantified using ImageJ software.

### 2.3 Glucose Production

Primary hepatocytes (250–300 K/well) were seeded overnight in a 12‐well plate and pre‐treated with salicylate or metformin for 3 h, Hepatocytes were washed twice in warm PBS prior to their medium was changed to glucose production medium containing DMEM glucose‐free pyruvate (2 mM), lactic acid (10 mM), glutamine (4 mM) with or without 100 nM of glucagon, with salicylate doses of 1, 2, 5 mM and metformin 0.5 mM. Cells were then incubated for 12 h, and 0.5 mL of medium was collected and subjected to glucose assay using the Amplex Red Glucose/Glucose Oxidase Assay Kit as described by the company (Invitrogen, A22189). The amount of glucose was then normalized to protein content. We did not measure glycogenolysis; however, previous studies have found that within 8–9 h of culturing, cultured hepatocytes are depleted of glycogen [16].

### 2.4 Analysis of Mitochondrial Respiration

The Seahorse XF24e analyzer was used to analyze the mitochondrial respiration of WT and KI hepatocytes. Cells (3 × 10^4^/well) were seeded on gelatin‐coated XF‐24 well plates to assess the effect of salicylate. In these experiments, the real‐time effect of salicylate on mitochondrial energetics was assessed by injecting the salicylate dose indicated in the figure legends to evaluate its effect on basal mitochondrial OCR. The ATP‐linked respiration, H^+^ (proton) leak, maximal respiratory capacity, and non‐mitochondrial respiration were determined using modulators of cellular respiration (i.e., oligomycin, FCCP, rotenone, and antimycin). Cellular respiration was normalized to protein content/well. The various mitochondrial parameters were normalized to protein content/well within the Seahorse plate.

### 2.5 Measurement of ATP, ADP and AMP

Frozen tissue (50–100 mg) was ground to a fine powder under liquid nitrogen, using a pestle and mortar. The tissue was transferred to microcentrifuge tubes and resuspended in 300 μL of 1 M ice‐cold perchloric acid (PCA) tissue, then vortexed for 30 s. The sample was further homogenized on ice using a Dounce homogenizer. Samples were centrifuged for 3 min at 12900 rpm at 4°C, and 100 μL of the supernatant was taken into a fresh tube, prior to neutralization by adding 60 μL of chilled 2.3 M KHCO_3_. Samples were then vortexed and left on ice for 2 min. Tubes were then centrifuged for 3 min at 12900 rpm at 40°C to remove potassium perchlorate. Levels of AMP, ADP, and ATP were measured using a TSQ Quantiva interfaced with an Ultimate 3000 Liquid Chromatography system (ThermoScientific), as described by [17] with modification of the LC gradient. The porous graphitic carbon column (HyperCarb 30 × 1 mm ID 3 mm; Part No: C‐35003‐031030, Thermo‐Scientific) was maintained at a constant temperature of 42°C and equilibrated for 11 min with 7% buffer B at a constant flow rate of 0.045 mL/min. Aliquots of 1 μL of each sample were loaded onto the column, and the three nucleotides were eluted with a linear gradient of 7%–12% buffer B over 3 min, then from 12% to 90% buffer B within 2 min, and finally from 90% to 100% buffer B within 5 min. The Quantiva parameters and LC buffers were maintained as described earlier [17].

### 2.6 FBP1 Activity Assay

FBP1 activity in mouse primary hepatocytes was determined in vitro by monitoring the formation of F6P using a coupled spectrophotometric assay as previously described using 1.5 μg lysates [12]. FBP1 expression was assessed by immunoblotting using an antibody from ProteinTech (12842‐1‐AP) and Vinculin (Cell Signaling Technology, 13901‐T) as a loading control.

### 2.7 Proteomics

Hepatocytes from 4 mice of both genotypes were cultured in 22 cm^2^ dishes and subjected to DIA Proteomics, which was carried out essentially as described previously [18, 19, 20, 21]. Briefly, prior to proteomic analysis, cells were lysed with 0.5 mL proteomics lysis buffer (4% SDS, 50 mM, Triethylammonium bicarbonate (TEAB) pH 8 and TCEP 10 mM). Samples were loaded into protein loBind tubes (Eppendorf) then boiled in a mixing shaker (ThermoMixer) for 5 min at 95°C with 500 RPM. They were then sonicated (15 cycles of 30 s on/30 s off), using a Biorupter sonicator. Protein quantification was done with EZQ protein quantification kit (Invitrogen). Samples were alkylated with iodoacetamide (IAA), 20 mM at room temperature for 1 h in the dark prior to 1.2% of phosphoric acid being added with vortexing, to neutralize. Samples were diluted in a 1:7 ratio with S‐Trap binding buffer containing 100 mM TEAB pH 7.1, prepared in 90% HPLC methanol grade, prior to loading the samples into S‐Trap mini columns and centrifuged at 4000 g for 30 s. The columns were then washed 5 times with S‐Trap binding buffer.

Trypsin digestion was carried out. The digestion mixture contained 50 mM ammonium bicarbonate with trypsin diluted 1:20 trypsin: protein ratio, added on top of the column and incubated at 47°C for 2 h. Peptides were collected by centrifugation at 4000 g after adding 80 mL of digestion buffer, repeated twice using 80 mL of 0.2% formic acid and 80 mL of 50% acetonitrile containing 0.2% formic acid. Collected peptides were dried using a Speed vac (GeneVac), then reconstituted in 1% formic acid by incubating tubes on a shaker mixer at 30°C for 1 h with shaking at 1000 rpm. Peptides were analyzed on a Q Exactive plus, Mass Spectrometer (Thermo Scientific). LC–MS was carried out as described previously [18]. Data are available via ProteomeXchange with identifier PXD053975.

### 2.8 Mass Spectrometry Data Analysis

Raw mass spectrometry data was processed using Spectronaut (Biognosys) version 15.0.210615.50606 with the DirectDIA option selected. Briefly, these parameters were used for the processing: cleavage rules were set to Trypsin/P, maximum peptide length 52 amino acids, minimum peptide length 7 amino acids, maximum missed cleavages 2. Carbamidomethylation of cysteine was set as a fixed modification, with the following variable modifications selected: oxidation of methionine, deamidation of asparagine and glutamine, and acetylation of the protein N‐terminus. The FDR threshold for both precursor and protein was set to 1%. Profiling and imputation were disabled. Quant 2.0 was selected. DirectDIA data were searched against a rat database from Uniprot release 2020 06. Estimates of protein copy number per cell were calculated using the histone ruler method [22].

### 2.9 Experimental Animals

C57BL/6J and NTac FBP1^G27P^ (FBP1 KI) mice were bred (Het × Het) to generate FBP1 KI homozygous and wild‐type littermate control (Control) as described [12]. Animals were group‐housed (maximum 4/cage) and maintained in a standard temperature and humidity‐controlled environment on a 12/12 h light/dark cycle with *ad libitum* access to water and standard chow (RM1; SDS diets UK) and with environmental enrichment (twizzle bedding, house, nestlet, chew stick). All animal procedures were approved by the University of Dundee Ethical Review Process and performed following UK Home Office Regulations (Project Licenses PC4E8729F, PP2397307). For tissue studies, male and female animals between 6 and 12 months were used.

### 2.10 Experimental Groups

Male FBP1 KI and Control mice between 12 and 14 weeks of age were placed on a high fat diet (HFD; 824 053 containing 45% AFE fat lard; Special Diet Services, Witham, UK) for 14 weeks to induce a pre‐diabetic insulin resistant state (Figure 2A; [23]). After 14 weeks on HFD diet, half of the animals were randomly allocated, with the investigator blinded, to a high fat diet supplemented with salicylate (SA; 3 g/kg) for a further 6 weeks, generating 4 experimental groups: (i) Control, (ii) Control + SA, (iii) KI, and (iv) KI + SA (*n* = 8–10 per group). Based on typical consumption of 2.9 g of chow a day, this achieved oral dosing of approximately 190 mg/kg per day, similar to the higher levels of salicylate used to observe antihyperglycaemic effects in human studies [1, 24, 25]. Sample size was arrived at following a power calculation based on previous work [26].

### 2.11 Metabolic Phenotyping

For a schematic timeline, see Figure 2A. Body weight was measured weekly, and body composition was assessed at 9 am using an EchoMRI machine (EchoMRI Texas, USA) at baseline (week 0), after 12 weeks on HFD, and at week 18 after four weeks on their respective diets. Tolerance tests occurred during the day, with fasting beginning at 8 am. Oral glucose tolerance tests (oGTT) were performed at weeks 4, 12 and 16. Animals were fasted for 4 h, and blood glucose was measured from the tail vein using a glucometer (Bayer, UK). Animals were given a 50 mg glucose bolus by oral gavage, and blood glucose was measured from the tail vein at time points indicated in the figures. For oral glucose insulin‐stimulated secretion (oGSIS) assessments, a small blood sample was taken from the tail vein at 0, 3‐, 15‐, and 30‐min intervals following a 50 mg oral glucose bolus, as described for the oGTT. Plasma insulin contents were measured by enzyme‐linked immunosorbent assay (ELISA; Crystal Chem, Elk Grove Village, IL, USA) according to the manufacturer's instructions. A pyruvate tolerance test (PTT) was performed at week 18. Following a 4‐h fast, a basal blood glucose measurement was taken from the tail vein. Animals then received pyruvate (Sigma made up in sterile water; 1 g/kg *i.p*.), and blood glucose was re‐measured at 30, 60‐, and 120 min.

At the study's termination, animals fasted for 4 h prior to insulin stimulation (3 U/kg *i.p*.). Animals were sacrificed by cervical dislocation, and tissue was dissected and either flash frozen in liquid nitrogen or fixed in PFA (4%). All data are presented as mean ± SEM. Statistical analysis was performed using SPSS (IBM v29 with PROCESS 4.3). Individual animals were investigated as the experimental unit. There were no adverse events.

### 2.12 Histology

Portions of livers from both genotypes of mice, fed either a HFD or an HFD supplemented with salicylate, were fixed in freshly prepared 4% paraformaldehyde in 0.1 M PBS (pH 7.4) overnight. The tissues were then embedded in paraffin and sectioned into 5 μm thick sections. The sections were stained using the hematoxylin and eosin (H&E) procedure. Another portion of the liver was frozen in dry ice and sectioned at a thickness of 5 μm using a cryostat. Lipid droplets in these sections were stained with Oil Red O, following the method of Lillie and Ashburn [27]. The H&E and Oil Red O slides were scanned in brightfield mode using a Plan‐Apochromat 20×/0.8 objective; using the slide scanner is a Zeiss Axioscan. Images scanned were analyzed to quantify the lipid droplets throughout the entire section using QuPath 0.4.3 software.

### 2.13 Statistical Analysis

For statistical testing of two groups, except proteomics, unpaired *T*‐tests were carried out. For statistical testing of three or more groups, ANOVA was carried out followed by post hoc test in GraphPad Prism. For proteomics, we applied False Discovery Rate correction to the empirical Bayes test in the limma R package, with significant results determined based on *q*‐values. Correlation Adjusted Mean Rank Analysis (CAMERA) was carried out on expressed gene sets for glycolysis and TCA cycle within the limma pipeline. All data are presented as the mean ± SEM. Differences were considered statistically significant if *p* value or *q* value was < 0.05. Heat maps were generated using the Morpheus tool from the Broad Institute (http://software.broadinstitute.org/morpheus). For Gene Ontology (GO) Term enrichment analysis, GO terms enriched in proteins with statistically significant changes in expression were identified using the functional annotation tools within DAVID Bioinformatics Resources 6.8, NIAID/NIH (https://david.ncifcrf.gov/). Experimental and control groups are identified in each figure. An experimental unit was a cell well. Experiments were carried out on at least three experimental units as described in the figure legends. Cell wells were allocated randomly to treatment groups.

## 3 Results

### 3.1 Evidence That Salicylate Acts on FBP1, Not AMPK, nor mTOR Signaling, to Regulate Glucose Production in Hepatocytes

**FIGURE 1:**
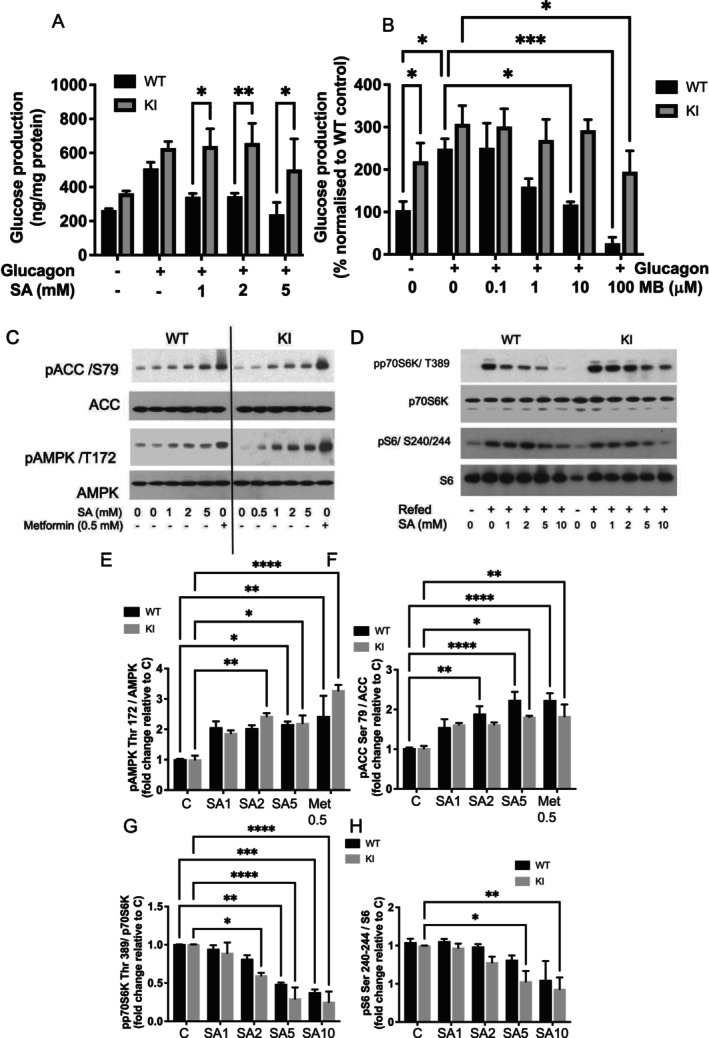
Glucose production and cell signaling in hepatocytes extracted from WT and KI animals. (A, B) Liver cells pre‐treated with salicylate (SA, A) or MB05032 (MB, B) were placed in glucose‐free medium for 12 h in the presence or absence of glucagon (100 ng/mL) and glucose production determined, then presented in bar graphs *N* = 4. (C–H) Liver cells were pre‐treated with salicylate or metformin doses shown for 1 h in minimal medium, prior to lysis and western blot analysis for AMPK signaling (C) and mTOR signaling (D), and then quantified (E–H) *N* = 4. Representative western blots are shown and data quantified across repeat blots is presented in bar graphs. Two‐way ANOVA was performed with Fisher LSD test (glucose) or Dunnett (blots) post hoc test, **** denotes *p* < 0.0001, *** denotes *p* < 0.001, ** denotes *p* < 0.01, * denotes *p* < 0.05.

Prior to carrying out an in vivo dietary study, we compared properties of salicylate in isolated hepatocytes extracted from FBP1 KI mice and their WT littermates from heterozygous matings. In these experiments, we discovered that glucagon‐stimulated hepatocyte glucose production was significantly suppressed in response to salicylate in WT cells (Figure 1A); in contrast, glucose production in KI hepatocytes was resistant to salicylate (Figure 1A). Consistent with salicylate acting in a FBP1‐dependent manner indirectly through the AMP‐binding site, the results were qualitatively similar to those obtained with MB05032, which is an allosteric inhibitor of FBP1 [28] (Figure 1B). MB05032 inhibits FBP1 by binding to the AMP binding site, and the KI form of FBP1 has previously been shown to be resistant to the drug [12], consistent with our findings (Figure 1B). Basal glucose production in KI cells tended to be elevated compared with WT cells (Figure 1A,B). In cell signal transduction experiments, we found that regulation of AMPK signaling by salicylate doses (24 h treatment), measured by p‐AMPKα Thr172 and phosphorylation of its substrate ACC Ser79, was similar in both genotypes (Figure 1C,E,F). mTOR signaling, measured by p70S6K pThr389 and S6 pSer240/244, also was regulated similarly in both genotypes (Figure 1D,G,H). Metformin appears as a positive control in the AMPK‐signaling experiments.

### 3.2 Evidence That Antihyperglycaemic Effect of Salicylate in HFD Feeding Depends on Inhibition of FBP1

**FIGURE 2:**
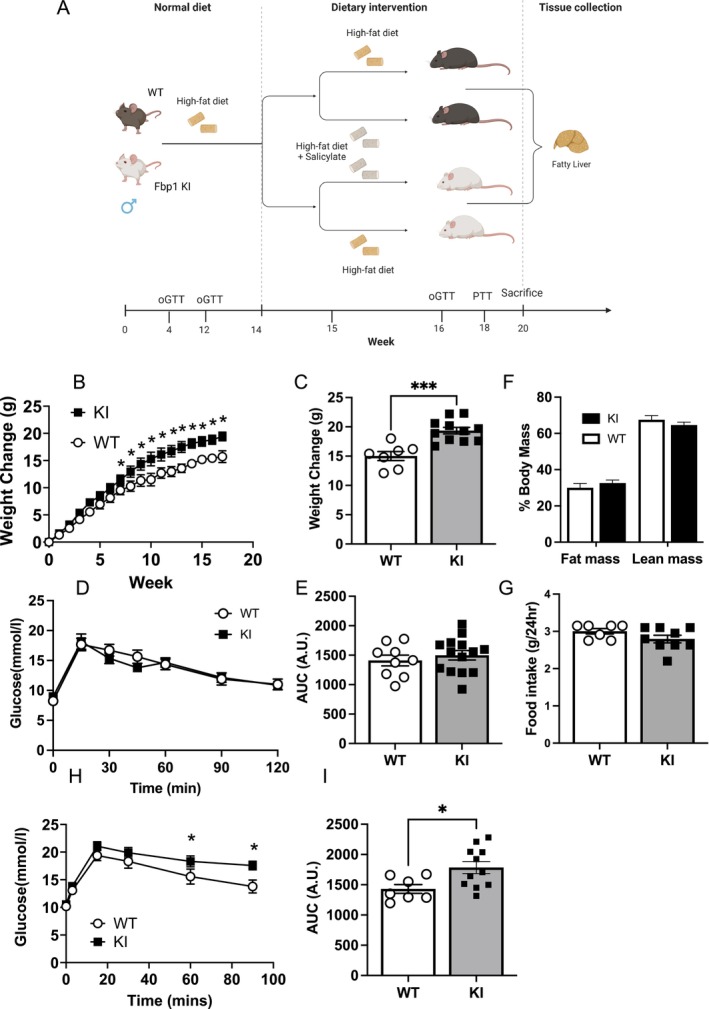
Response of WT and KI animals to High Fat Diet (HFD). (A) Schematic of study. (B, C) Weight change of animals. (D, E) Oral glucose tolerance (oGTT) tests were carried out after 4 weeks on HFD, and area under the curve (AUC) determined. (F) Echo MRI analysis of fat and lean mass prior to initiation of diet. (G) Food intake was monitored over a 24‐h period. (H, I) Glucose tolerance tests were carried out after 12 weeks on HFD, and area under the curve (AUC) determined. Individual mice are depicted by the symbols in each bar graph or mean ± SEM in graphs. Statistically significant differences are shown after two‐way unpaired *T*‐test; *** denotes *p* < 0.001, * denotes *p* < 0.05.

To investigate whether FBP1 is required for the antihyperglycaemic action of salicylate in vivo, we fed WT and KI mice a HFD for 14 weeks (schematic, Figure 2A). Comparison of the animals prior to salicylate treatment is shown in Figure 2. There was no significant difference in body weight between WT and KI mice up to 8 weeks on HFD, consistent with a previous study [12]. KI animals put on more weight, notably after week 9–10 (Figure 2B,C); however, glucose tolerance and body composition (lean and fat mass) were similar between genotypes after 4 weeks on HFD (Figure 2D–F). Food intake was unchanged (Figure 2G). Consistent with their tendency to gain more fat after 12 weeks on HFD, KI mice were less able than WT to clear an oral glucose load (Figure 2H,I).

**FIGURE 3:**
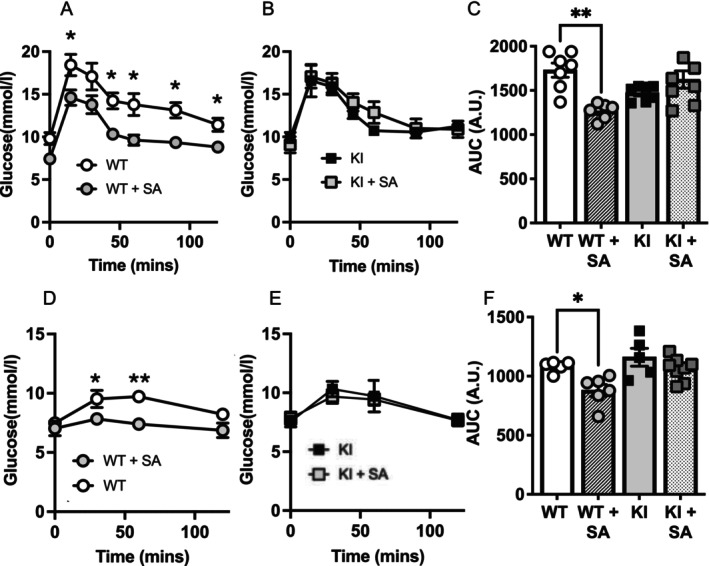
Tolerance test responses in WT and KI animals. (A–C) Glucose tolerance tests were carried out after 2 weeks on salicylate in WT (A) and KI (B) animals, and area under the curve (AUC) determined, (C). (D–F) Pyruvate tolerance (PTT) tests were carried out after 4 weeks on salicylate in WT (D) and KI (E) animals, and area under the curve (AUC) determined, (F). Individual mice are depicted by symbols in each bar graph or mean ± SEM in graphs. Statistically significant differences are shown after one‐way ANOVA and Tukey post hoc test, ** denotes *p* < 0.01, * denotes *p* < 0.05.

After the HFD run‐in period, half of the animals were switched on to a HFD supplemented with (5 g/kg) salicylate until sacrifice, whilst the remainder remained on the original HFD. The earlier differences in glucose intolerance due to genotype became attenuated as the HFD exposure continued (Figure 3A,B). Regarding salicylate intervention, glucose tolerance of WT but not KI littermates improved after salicylate treatment (Figure 3A–C).

### 3.3 Pyruvate Fate In Vivo Indicates Salicylate Acts on Glucose Production Through FBP1

The results so far are consistent with salicylate suppressing glucose production in a manner dependent on the FBP1 nucleotide binding site. To provide more evidence for this mechanism, we carried out pyruvate tolerance tests (PTT) in vivo and supporting cell culture investigations. In the PTTs, glucose produced by WT animals was lower in salicylate‐treated animals compared with HFD‐treated animals (Figure 3D,F). In contrast, KI animals were resistant to salicylate, with no significant difference between salicylate‐fed and HFD‐fed KI animals (Figure 3E,F). These findings suggest that the KI mouse is resistant to the effects of salicylate on gluconeogenesis.

**FIGURE 4:**
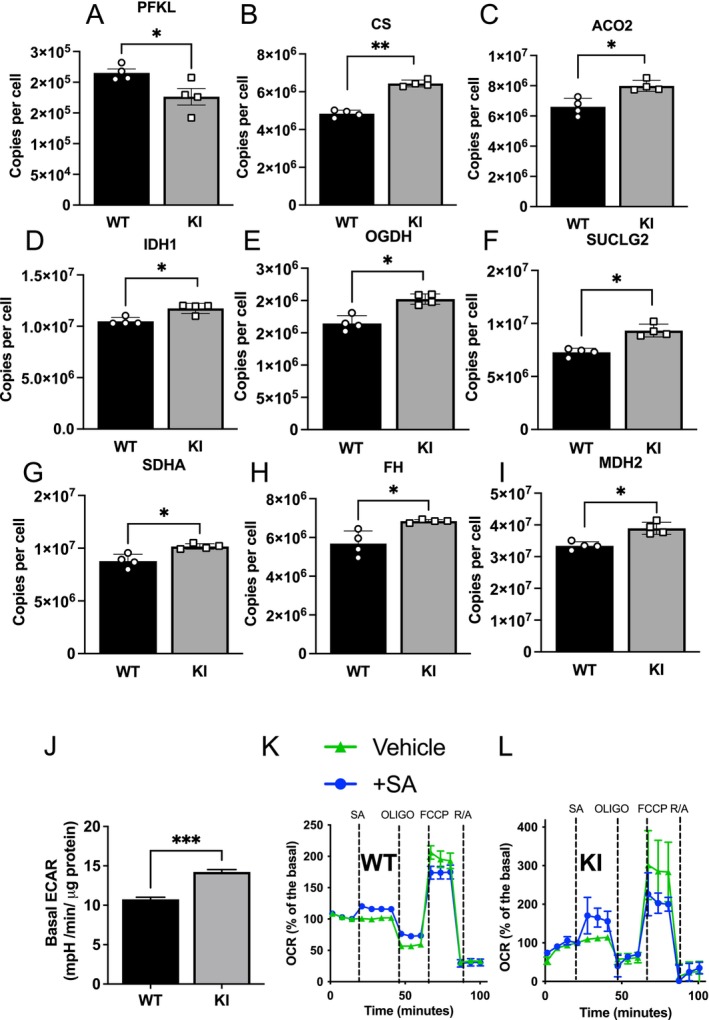
Proteomic analysis of liver cells. Wild type and KI liver cells were subject to proteomics to compare protein expression in the two genotypes. (A) The glycolytic protein PFKL, (B–I) TCA enzymes whose expression is significantly different between genotypes (J) Quantitation of basal ECAR in WT and KI cells. (K, L) Oxygen consumption rate during a respiratory control experiment was determined by Seahorse. Oligomycin (oligo), carbonylcyanide‐p‐trifluoromethoxyphenylhydrazone (FCCP) and rotenone (R) were each used at 1 μM, and Antimycin A (‘A’) was used at 2 μM. *N* = 3. Individual mice are depicted by symbols in each bar graph or mean ± SEM in graphs. Significant differences are shown based on *q*‐value adjustment after limma, except for t‐test in (J); *** denotes *p* < 0.001,** denotes *p* < 0.01, * denotes *p* < 0.05.

In isolated hepatocytes, proteomics analysis in WT and KI cells identified a tendency for glycolytic enzyme expression to be reduced in KI hepatocytes. Correlation Adjusted Mean Rank Analysis (CAMERA) analysis of 20 manually curated glycolytic enzymes found that this set was significantly reduced (*p* = 7.76E‐06), with phosphofructokinase PFKL also individually significantly reduced (Figure 4A). Despite these adaptations, basal ECAR (a measure of glycolysis) was higher in KI cells than in WT cells (Figure 4J). CAMERA analysis also determined that a manually curated set of 21 TCA enzymes was upregulated (*p* = 0.001047) and in addition, individual enzymes were significantly upregulated, including TCA citrate synthase (CS, Figure 4B), aconitase 2 (ACO2, Figure 4C), isocitrate dehydrogenase 1 (IDH1, Figure 4D), oxoglutarate dehydrogenase (OGDH, Figure 4E), succinyl‐CoA ligase 2 SUCLG2 (Figure 4F), succinate dehydrogenase A (SDHA, Figure 4G), fumarate hydratase (FH, Figure 4H), malate dehydrogenase (MDH2, Figure 4I) in KI livers compared with WT livers. These results are suggestive of enhanced oxidative disposal of pyruvate, possibly to restrict gluconeogenesis and futile cycling. Despite these adaptations, TCA cycle activity, measured by OCR, was similar and salicylate raised OCR in both genotypes (Figure 4K,L).

### 3.4 Role of FBP1 in Effects of Salicylate on Body Weight, Energy Levels, and Lipid Storage

**FIGURE 5:**
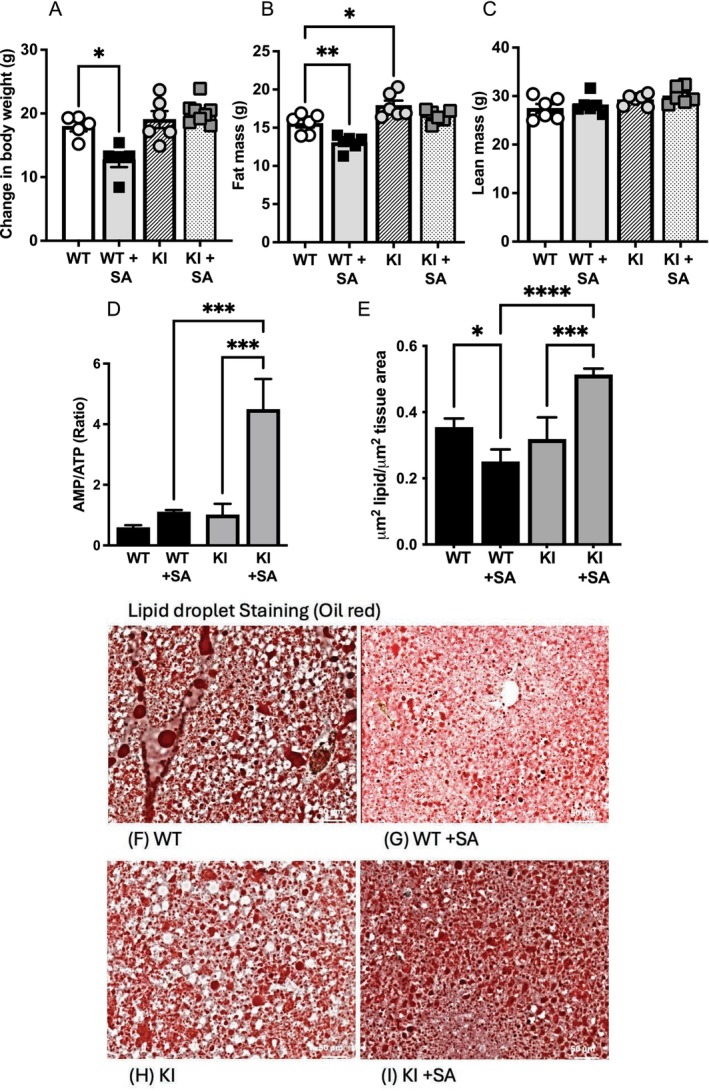
Effect of dietary salicylate on fat storage and body weight. (A–C) Changes in body weight and fat/lean mass detected by Echo MRI, following inclusion of salicylate in the diet. (D) Nucleotide levels were determined by LCMS and the AMP/ATP ratio is presented. (E–I) Lipid content was determined from liver tissue slices using oil red O stain and quantified (E). Representative images for each treatment are shown (F–I). Individual mice are depicted by symbols in each bar graph for in vivo studies; other data is presented as mean ± SEM and representative microscope images in F–I. For the other experiments, *N* = 4. Statistically significant differences are shown after one‐way ANOVA and Tukey post hoc test; **** denotes *p* < 0.0001, *** denotes *p* < 0.001, ** denotes *p* < 0.01, * denotes *p* < 0.05.

Consistent with previous findings, we found that salicylate reduced body weight in WT animals on HFD (Figure 5A). This weight loss was primarily due to a significant reduction in fat mass in WT animals on salicylate (Figure 5B). In contrast, there was no effect of salicylate on lean or fat mass in KI animals. KI mice had more fat mass than WT mice (Figure 5B,C) and this was unchanged by salicylate. Two weeks of salicylate in the diet raised AMP/ATP ratios in tissues from livers harvested from mice of either genotype (Figure 5D); however, this was much more pronounced in KI livers. In microscopy analysis, we also documented a change in lipid storage, finding that salicylate reduced lipid storage in WT livers but not in KI livers (Figure 5E–I). To investigate this difference further, we studied a manually curated list of 18 genes previously identified as being critical mitochondrial fatty acid oxidation enzymes [29]. CAMERA analysis found that this list was upregulated in KI livers compared with WT (*p* = 0.000104). Twelve of the genes were significantly upregulated, including medium‐chain 3‐ketoacyl‐CoA thiolase (ACAA2), long‐chain acyl‐CoA dehydrogenase (ACADL), medium‐chain acyl‐CoA dehydrogenase (ACADM), short‐chain acyl CoA dehydrogenase (ACADS), carnitine palmitoyltransferase 2 (CPT2), 2,4‐Dienoyl‐CoA reductase (DECR1), Δ3,5‐Δ2,4‐Dienoyl‐CoA isomerase (ECH1), short‐chain enoyl‐CoA hydratase (ECHS1) enoyl CoA delta‐isomerase 1 and 2 (ECI1, ECI2), mitochondrial trifunctional protein, beta subunit (HADHB) and Mitochondrial Carnitine/Acylcarnitine Carrier Protein SLC25A20 (Figure S1). Besides mitochondrial oxidation, fatty acids in hepatocytes may also be directed to peroxisomal oxidation, or they may be exported, and we found evidence of upregulation of these pathways too in the KI genotype. Four peroxisomal proteins, including the rate‐determining enzyme acyl coenzyme A oxidase 1 (ACOX1) [30], peroxisomal targeting signal 1 receptor (PEX5), peroxisomal biogenesis factor 11A (PEX11A) and hydroxysteroid beta hydrogenase 17B4 (HSD17B4), were upregulated in KI livers compared to WT (Figure S1). PEX5 mediates uptake of β‐oxidation enzymes into peroxisomes [31] and PEX11A promotes peroxisomal biogenesis [32]. HSD17B4 catalyzes two of the four steps of fatty acid degradation in the peroxisome [33]. In addition, the lipid transporter MTTP was upregulated (Figure S1).

## 4 Discussion

In this study, we have evidenced genetic ablation of the antihyperglycaemic action of salicylate through a KI mutation that renders FBP1 insensitive to AMP indirectly generated by salicylate. On HFD, FBP1 KI mice were resistant to the effects of salicylate on glucose tolerance. In vivo PTTs provided strong evidence that salicylate suppresses hyperglycaemia in HFD through a FBP1‐dependent mechanism. The dose used is consistent with higher doses used in humans to achieve antihyperglycaemic effects rather than analgesic effects [1, 24, 25].

We carried out proteomic analysis in hepatocytes of differences in protein levels between the WT and FBP1 KI genotypes. We observed small changes in a variety of glycolytic and TCA enzymes; however, these were only significant for a small number of TCA enzymes and the glycolytic enzyme PFKL. We used follow‐up CAMERA analysis to study overall trends in pathways quantitatively, finding that the glycolytic pathway was upregulated and TCA cycle downregulated in KI hepatocytes compared with WT hepatocytes; however, these differences did not translate into significant functional changes.

In addition, whilst we observed upregulation of a wide range of mitochondrial fatty acid oxidation enzymes, we also noted upregulation of several peroxisomal oxidation genes, and the VLDL lipid export regulator MTTP, hinting at diversification of lipid disposal in the KI mice. Further work will be required to determine whether these changes contribute to the resistance of KI animals to the effects of salicylate on weight gain and hepatic lipid storage. Supporting cell studies allowed us to investigate the mechanism of salicylate on glucose metabolism in more detail. These results are consistent with salicylate acting on the mitochondria to increase AMP‐dependent signaling levels in both WT and KI genotypes. Salicylate raised the AMP/ATP ratio only modestly in WT liver tissue, consistent with previous data; however, there was a larger effect in the livers of KI mice [10], indicating that the mutation magnifies the effect of salicylate on AMP/ATP, although we were unable to detect corresponding changes in AMPK activation from tissues with different genotypes (data not shown). FBP1 protein expression levels were similar in both genotypes, and inhibition of FBP1 by salicylate was not detectable in a cell‐free assay, consistent with the inhibition being indirect, as might be mediated by a diffusible factor such as AMP.

The effect of salicylate on weight was present in WT and absent in KI animals in vivo, and neither did salicylate reduce fat storage in the liver in KI hepatocytes as efficiently as it did in WT hepatocytes. Further work will be required to understand these observations and to determine whether weight loss and beneficial effects on glucose homeostasis induced by more recently identified protonophores, such as BAM15 [34], depend on FBP1. Our novel insight that there may be at least two targets underlying antihyperglycaemic effects and weight loss with salicylate, namely, (i) energy loss through disruption of ATP production and (ii) inhibition of FBP1, might help to explain previous observations [35] of a poor correlation between the potency of a protonophore and its potency as an antihyperglycaemic agent, something that has been hard to explain if only one target is considered. Although AMPK is directly targeted by salicylate, mutation of the binding site does not alter the effect of the drug on glucose homeostasis, nor on fat accumulation in mice [10].

We do not exclude that salicylate might act through other enzymes in other tissues, such as for example to mediate skeletal muscle glucose uptake, in which tissue FBP1 is not expressed; however, our data indicate regulation of FBP1 is a key determinant of the effect of the drug on glucose production and weight loss. In addition, further work will be required to validate FBP1 as a target of salicylate in humans. Indeed, more research on the consequences of FBP1 inhibition in humans is needed. There is preclinical evidence in mice that FBP1 inhibition might also promote insulin secretion [36]. Other preclinical evidence suggests that FBP1 overexpression acts as a tumor suppressor [37]; however, the lack of tumorigenesis with salicylate suggests that allosteric inhibition is a way of inhibiting FBP1, without promoting tumor progression.

## 5 Conclusion

Our results indicate that mice with AMP‐insensitive FBP1 are resistant to the antidiabetic actions of salicylate in animals fed HFD. Taken together with previous findings on metformin, the accidental discovery on at least two independent occasions of diabetes drugs capable of promoting allosteric FBP1 inhibition suggests this enzyme is a highly promising target for rational drug discovery approaches to develop next‐generation type 2 diabetes drugs based on the highly efficacious properties of metformin and salicylate.

## Author Contributions

**Raid B. Nisr:** data curation, formal analysis, visualization, writing – review and editing, investigation, methodology. **Abdelmadjid Atrih:** investigation. **Erika J. Gutierrez Lara:** investigation, formal analysis, writing – review and editing. **Douglas Lamont:** methodology. **Katarzyna M. Luda:** investigation. **Rory J. McCrimmon:** writing – review and editing, funding acquisition. **Kei Sakamoto:** writing – review and editing, supervision, formal analysis. **Graham Rena:** funding acquisition, supervision, project administration, writing – review and editing, writing – original draft, formal analysis, conceptualization. **Alison D. McNeilly:** funding acquisition, supervision, project administration, writing – review and editing, formal analysis, investigation, conceptualization.

## Conflicts of Interest

The authors declare no conflicts of interest.

## Supporting information


Figure S1.
**FIGURE S2**.

Table S1.

## Data Availability

The data that support the findings of this study are openly available in PRIDE at https://www.ebi.ac.uk/pride/markdownpage/proteomexchange, reference number PXD053975.
