# Supplementary material for: The Ancient Drug Salicylate Indirectly Targets Fructose‐1,6‐Bisphosphatase to Suppress Liver Glucose Production in Diet‐Induced Obese Mice
Source: Acta Physiol (Oxf). 2025 May 22;241(6):e70058. doi: 10.1111/apha.70058 (PMC12096142; doi:10.1111/apha.70058)

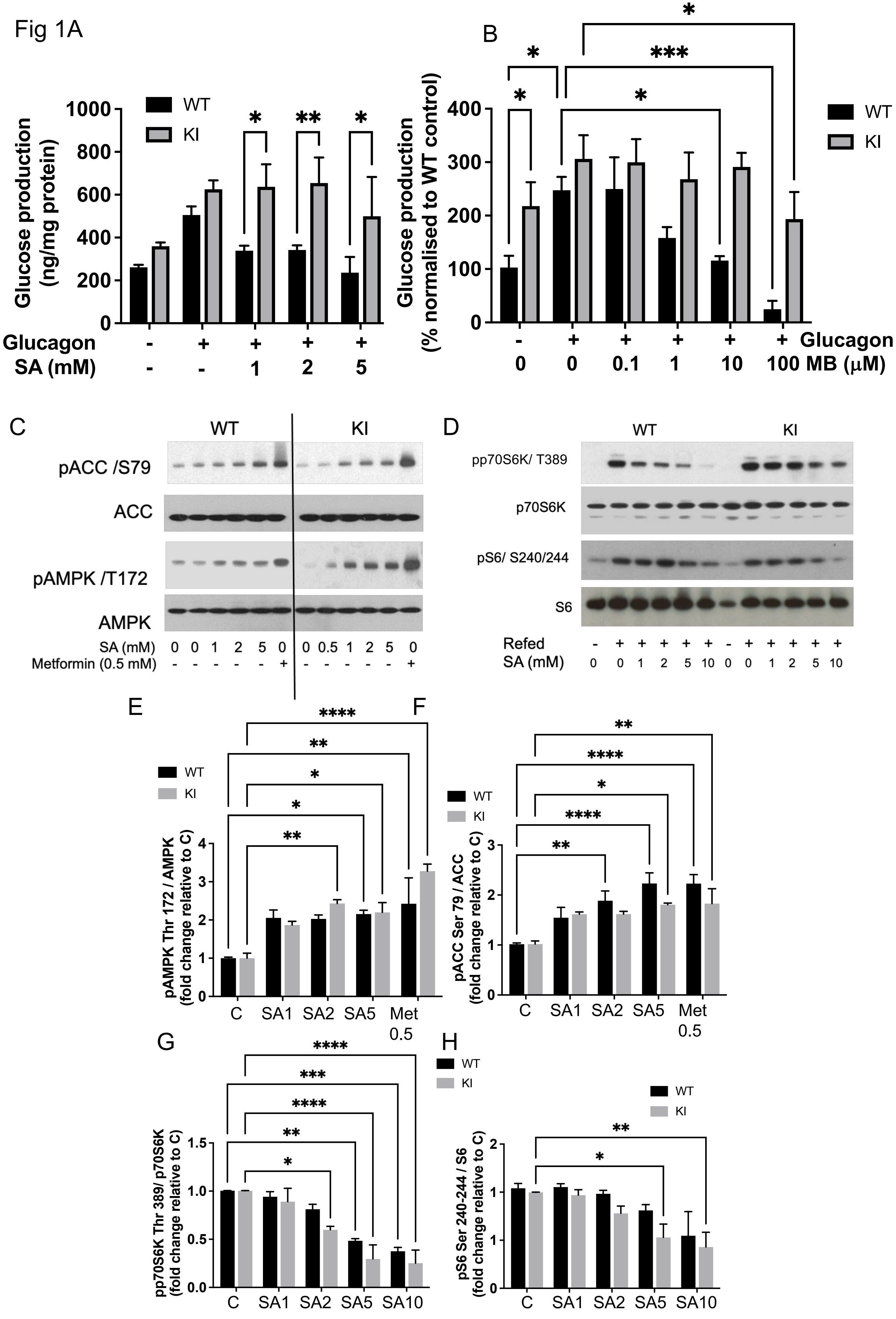
Fig 1A
Glucose production (ng/mg protein)
1000
800
600
400
200
0
WT
KI
*
**
*
Glucagon - + + + +
SA (mM) - - 1 2 5
B
Glucose production (% normalised to WT control)
400
300
200
100
0
*
*
***
*
*
WT
KI
- + + + + + Glucagon
0 0 0.1 1 10 100 MB (μM)
C
WT
KI
pACC /S79
ACC
pAMPK /T172
AMPK
SA (mM) 0 0 1 2 5 0 0 0.5 1 2 5 0
Metformin (0.5 mM) - - - - - + - - - - - +
D
WT
KI
pp70S6K/ T389
p70S6K
pS6/ S240/244
S6
Refed - + + + + + - + + + + +
SA (mM) 0 0 1 2 5 10 0 0 1 2 5 10
E
****
**
*
*
**
WT
KI
pAMPK Thr 172 / AMPK (fold change relative to C)
4
3
2
1
0
C
SA1
SA2
SA5
Met 0.5
F
**
****
*
****
**
WT
KI
pACC Ser 79 / ACC (fold change relative to C)
3
2
1
0
C
SA1
SA2
SA5
Met 0.5
G
****
***
****
**
*
pp70S6K Thr 389/ p70S6K (fold change relative to C)
1.5
1.0
0.5
0.0
C
SA1
SA2
SA5
SA10
H
WT
KI
WT
KI
**
*
pS6 Ser 240-244 / S6 (fold change relative to C)
2
1
1
0
C
SA1
SA2
SA5
SA10

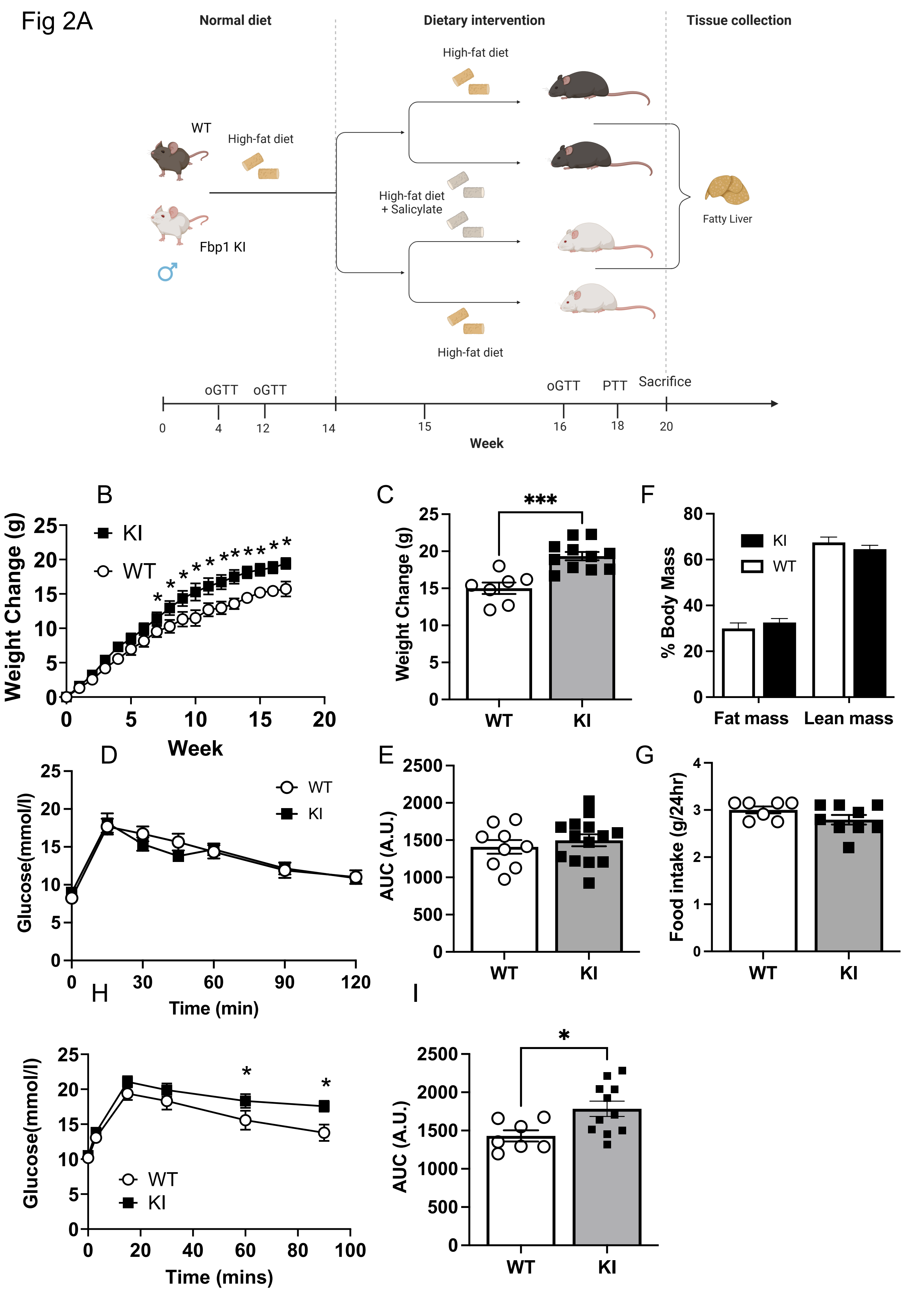

Fig 2A
Normal diet
Dietary intervention
Tissue collection
WT
High-fat diet
Fbp1 KI
High-fat diet
High-fat diet
+ Salicylate
High-fat diet
Fatty Liver
oGTT
oGTT
oGTT
PTT
Sacrifice
0
4
12
14
15
16
18
20
Week
B
Weight Change (g)
KI
WT
Week
C
Weight Change (g)
***
WT
KI
F
% Body Mass
KI
WT
Fat mass
Lean mass
D
Glucose(mmol/l)
WT
KI
Time (min)
E
AUC (A.U.)
WT
KI
G
Food intake (g/24hr)
WT
KI
H
Glucose(mmol/l)
WT
KI
Time (mins)
I
AUC (A.U.)
*
WT
KI

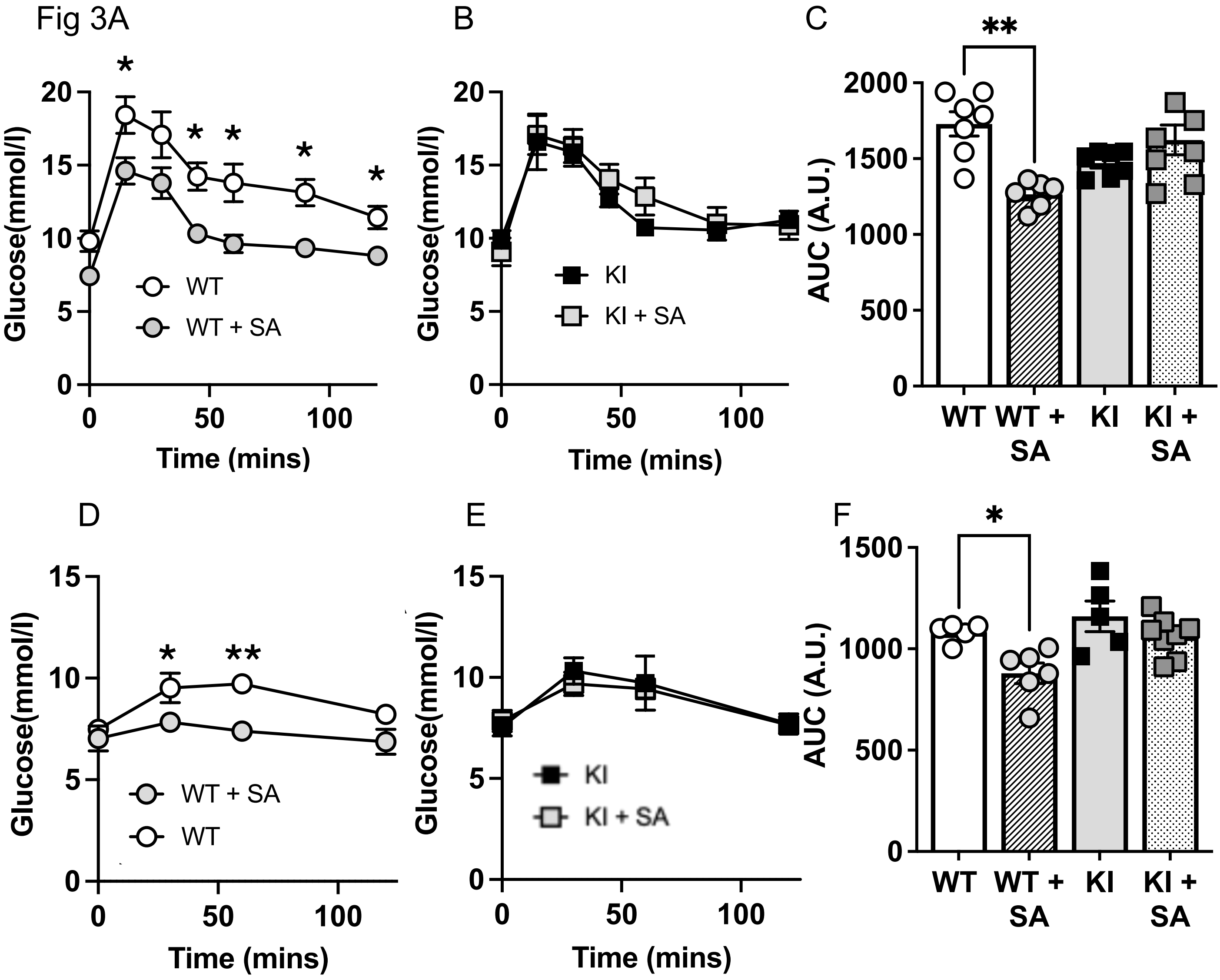

Fig 3A
Glucose(mmol/l)
Time (mins)
WT
WT + SA
B
KI
KI + SA
C
AUC (A.U.)
WT
WT + SA
KI
KI + SA
D
E
F

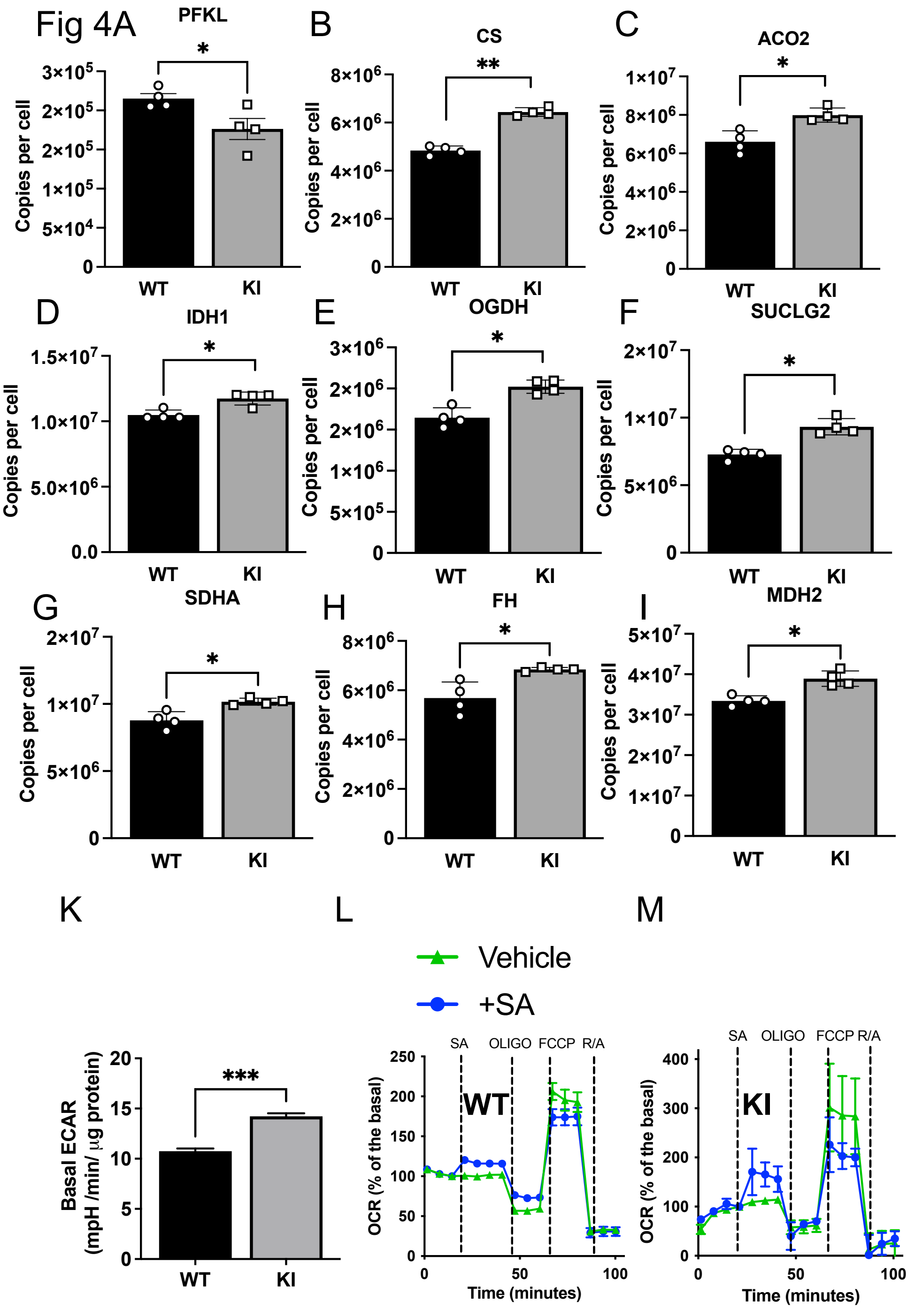
Fig 4A
PFKL
*
3×10^5
2×10^5
2×10^5
1×10^5
5×10^4
0
Copies per cell
WT
KI
B
CS
**
8×10^6
6×10^6
4×10^6
2×10^6
0
Copies per cell
WT
KI
C
ACO2
*
1×10^7
8×10^6
6×10^6
4×10^6
2×10^6
0
Copies per cell
WT
KI
D
IDH1
*
1.5×10^7
1.0×10^7
5.0×10^6
0.0
Copies per cell
WT
KI
E
OGDH
*
3×10^6
2×10^6
2×10^6
1×10^6
5×10^5
0
Copies per cell
WT
KI
F
SUCLG2
*
2×10^7
1×10^7
5×10^6
0
Copies per cell
WT
KI
G
SDHA
*
2×10^7
1×10^7
5×10^6
0
Copies per cell
WT
KI
H
FH
*
8×10^6
6×10^6
4×10^6
2×10^6
0
Copies per cell
WT
KI
I
MDH2
*
5×10^7
4×10^7
3×10^7
2×10^7
1×10^7
0
Copies per cell
WT
KI
K
L
M
Vehicle
+SA
Basal ECAR
(mpH /min/ μg protein)
***
20
15
10
5
0
WT
KI
SA
OLIGO
FCCP
R/A
WT
OCR (% of the basal)
250
200
150
100
50
0
0
50
100
Time (minutes)
SA
OLIGO
FCCP R/A
KI
OCR (% of the basal)
400
300
200
100
0
0
50
100
Time (minutes)

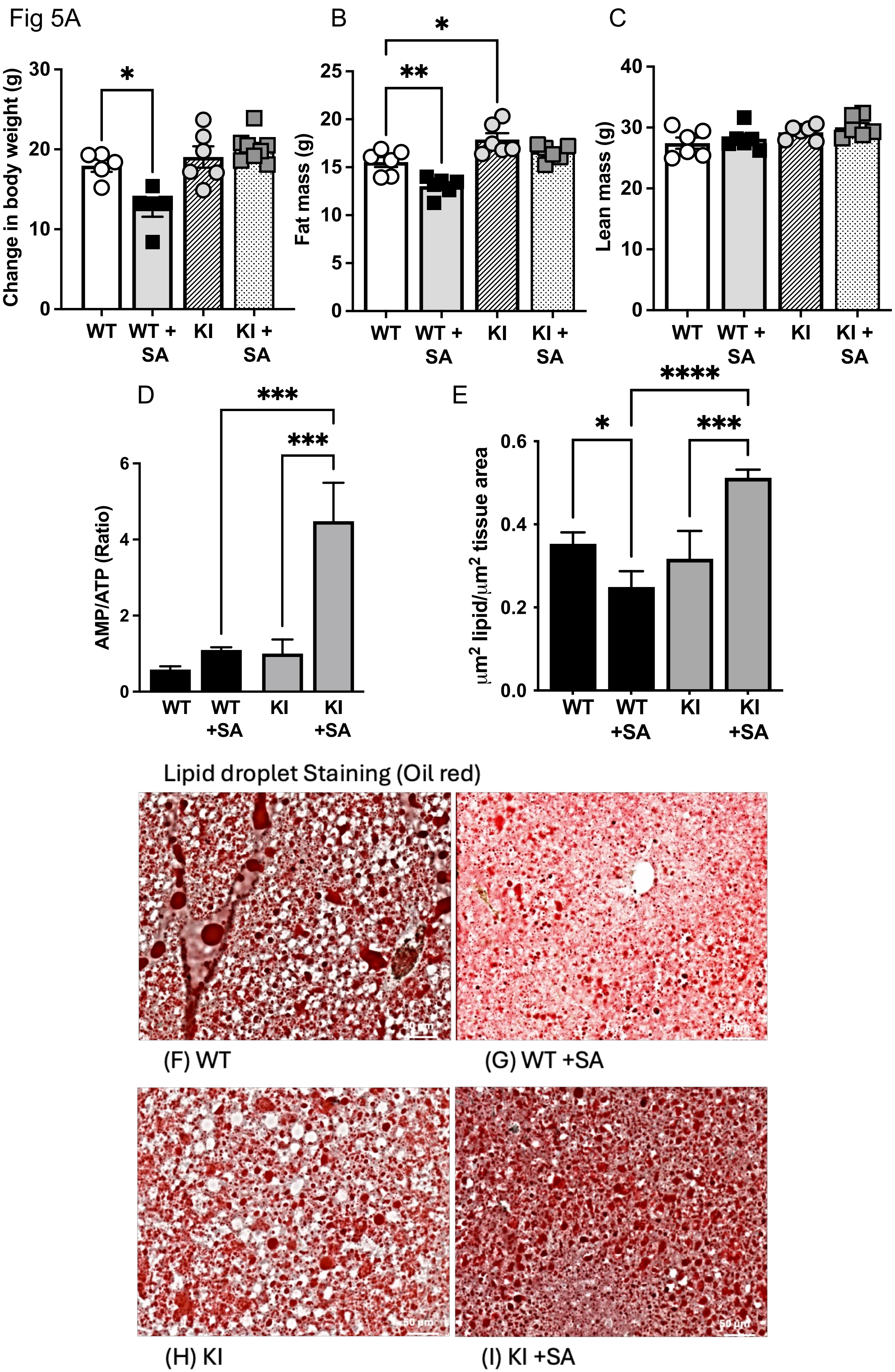
Fig 5A
Change in body weight (g)
30
20
10
0
*
WT
WT + SA
KI
KI + SA
B
Fat mass (g)
25
20
15
10
5
0
*
**
WT
WT + SA
KI
KI + SA
C
Lean mass (g)
40
30
20
10
0
WT
WT + SA
KI
KI + SA
D
AMP/ATP (Ratio)
6
4
2
0
***
***
WT
WT +SA
KI
KI +SA
E
μm² lipid/μm² tissue area
0.6
0.4
0.2
0.0
****
*
***
WT
WT +SA
KI
KI +SA
Lipid droplet Staining (Oil red)
(F) WT
(G) WT +SA
(H) KI
(I) KI +SA

Supplementary Figure 1

Fatty acid oxidation

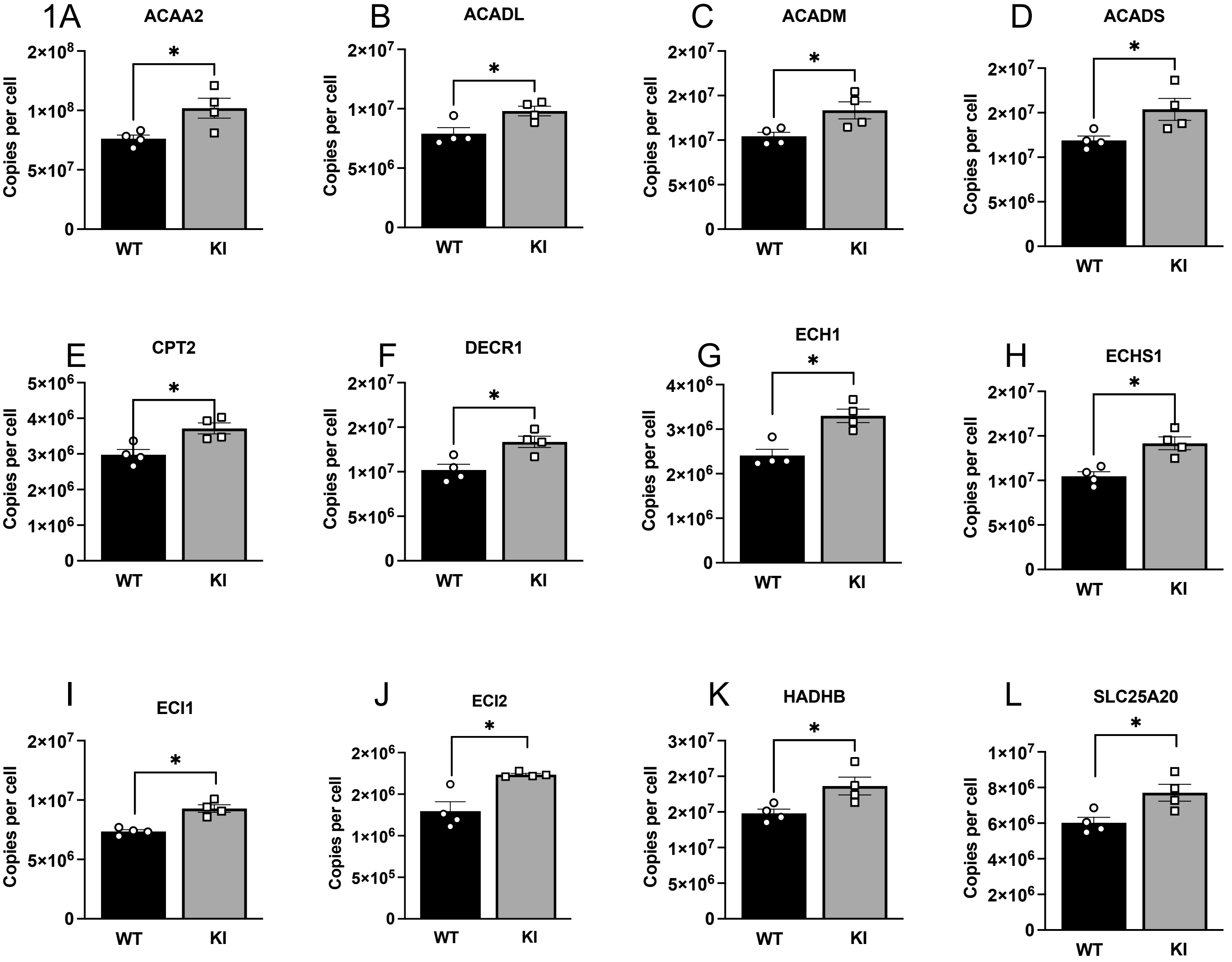

Peroxisomal oxidation

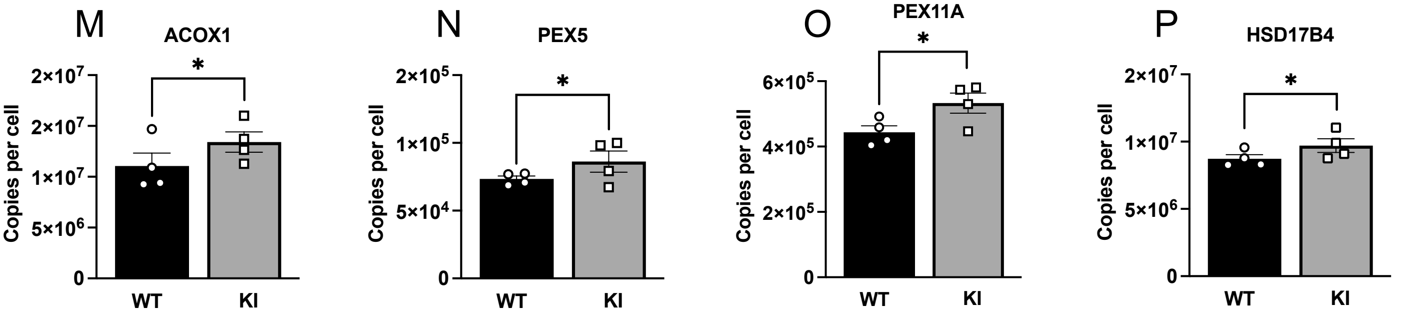

Transport

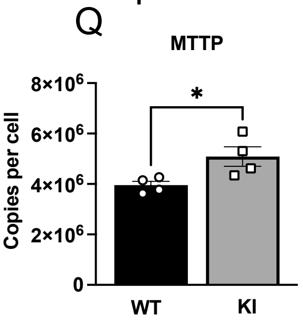

Supplementary Figure 2

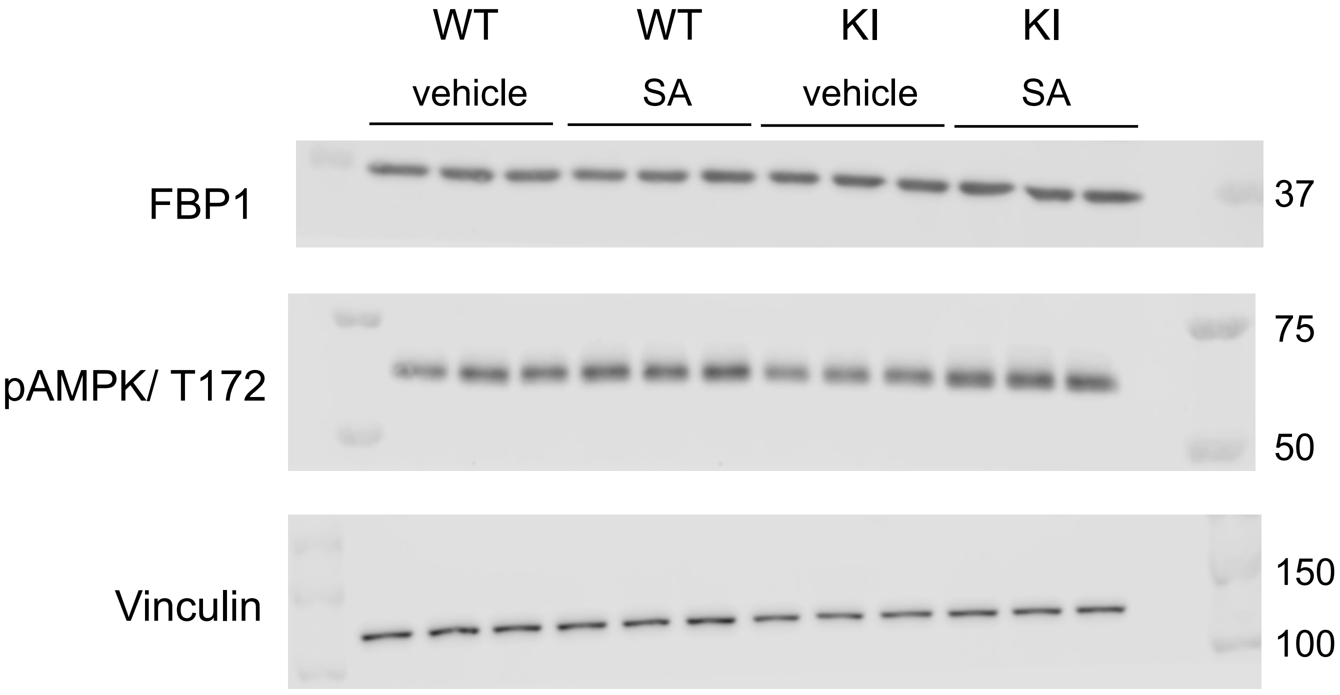

Supplement: Supplementary file 1 — Figure S1. FIGURE S2. [file APHA-241-e70058-s002.pdf]
