# Supplementary material for: The Ancient Drug Salicylate Indirectly Targets Fructose‐1,6‐Bisphosphatase to Suppress Liver Glucose Production in Diet‐Induced Obese Mice
Source: Acta Physiol (Oxf). 2025 May 22;241(6):e70058. doi: 10.1111/apha.70058 (PMC12096142; doi:10.1111/apha.70058)
Supplement: Supplementary file 2 — Table S1. [file APHA-241-e70058-s001.docx]

**Supplementary Table 1**

| **Glycolytic enzymes** | **TCA cycle enzymes** | **Fatty Acid Metabolism enzymes** |
| --- | --- | --- |
| Hk1 | Cs | Acaa2 |
| Hkdc1 | Aco2 | Acad9 |
| Pfkm | Idh3a | Acadl |
| Pfkl | Idh3b | Acadm |
| Aldoa | Idh3g | Acads |
| Aldoc | Suclg1 | Acadvl |
| Tpi1 | Sucla2 | Acat1 |
| Gapdh | Sdhd | Cpt1a |
| Pgk1 | Sdha | Cpt2 |
| Pgm1 | Fh | Decr1 |
| Pgm2 | Mdh1 | Ech1 |
| Pgm3 | Ogdh | Echs1 |
| Eno1 | Pdhb | Eci1 |
| Pkm | Pdha1 | Eci2 |
| Pklr | Aco1 | Hadh |
| Ldhd | Idh1 | Hadha |
| Ldha | Idh2 | Hadhb |
| Gpi | Suclg2 | Slc25a20 |
| Pfkp | Sdhb |  |
| Pgam1 | Sdhc |  |
|  | Mdh2 |  |
